# Gender invariance in the relationship between social support and glycemic control

**DOI:** 10.1371/journal.pone.0285373

**Published:** 2023-05-08

**Authors:** Joni S. Williams, Rebekah J. Walker, Leonard E. Egede

**Affiliations:** 1 Division of General Internal Medicine, Department of Medicine, Medical College of Wisconsin, Milwaukee, Wisconsin, United States of America; 2 Center for Advancing Population Science, Medical College of Wisconsin, Milwaukee, Wisconsin, United States of America; Edith Cowan University, AUSTRALIA

## Abstract

**Background:**

Social support (SS) is important in diabetes self-management; however, little is known about how different types of SS influence diabetes outcomes in men and women with type 2 diabetes mellitus (T2DM). Therefore, the aims of this study were to investigate the relationships between types of SS and glycemic control and self-care behaviors and assess whether the relationships differ by gender.

**Methods:**

Cross-sectional study of 615 adults from two primary care clinics in the southeastern U.S. Outcomes were hemoglobin A1c (HbA1c) extracted from the medical records, and self-management behaviors (general diet, specific diet, exercise, blood glucose testing, foot care) measured using the Summary of Diabetes Self-Care Activities (SDSCA). Independent variable was SS (emotional/informational, tangible, affectionate, positive social interaction) measured using the Medical Outcomes Study (MOS) SS Scale. Structural equation modeling (SEM) was used to understand pathways between SS and glycemic control based on a theoretical model.

**Results:**

Tangible support was significantly associated with self-care (r = 0.16; p = 0.046) and affectionate support was marginally associated with glycemic control (r = 0.15; p = 0.08) for both men and women. Using SEM to test gender invariance, there was no statistically significant difference in the meaning of SS between men and women. However, unique invariances in responses occurred, including a stronger relationship between tangible support and self-care for women (r = 0.24; p = 0.061).

**Conclusions:**

Of the four components of SS, tangible and affectionate support had the strongest influence on glycemic control. While affectionate support will improve glycemic control in both men and women, tangible support will improve self-care management, particularly in women.

## Introduction

Affecting more than 34 million adults in the United States [1], diabetes continues to be a significant cause of morbidity such as lower limb amputations, end-stage renal disease, and adult-onset blindness and mortality [1, 2]. Diabetes is the seventh leading cause of death with type 2 diabetes (T2DM) accounting for 90–95% of all diagnosed cases [1, 2]. Among adults 18 years of age and older, more than 7 million have undiagnosed diabetes, and approximately 35% have prediabetes based on their fasting blood glucose or hemoglobin A1c (HbA1c) level [1]. More than 7 million hospitalizations and 16 million emergency room visits have been associated with diabetes diagnoses, resulting in more than an estimated $327 billion in direct and indirect costs annually for medical care [1].

Achieving glycemic control requires a comprehensive approach to diabetes management that includes routine visits to providers along with adequate self-care such as eating a healthy diet, being physically active, adhering to prescribed medication plans, and monitoring blood glucose levels [3]. Daily self-management is essential for achieving optimal outcomes and preventing diabetes-related complications [3–6]; however, individuals often fail to reach targeted self-management goals [7], frequently lacking needed illness-specific support that has been shown to facilitate improved health outcomes [8, 9]. Social support–the perception of acceptance, care, provision, and assistance from certain individuals or groups of individuals, or the realization of actual support from another–has been shown to influence self-management, resulting in improved lifestyle behaviors, better clinical outcomes, and reduced psychosocial symptoms [7, 9–18]. In diabetes, social support has been considered a major component of self-management for achieving glycemic control, developing behaviors and skills to acquire healthier lifestyles, and improving outcomes [7, 10, 18]. It is associated with diagnosis acceptance, emotional adjustment, and reduced stress, and when perceived as satisfactory, protects against diabetes distress and burden [10, 14].

Evidence proposes different mechanisms for the relationship between social support and diabetes that are facilitated through the exchange of knowledge and information and assistance with access to resources [6]; however, the differential influences of social support by type and gender are still unclear. Previous research does suggest, however, that the influence of social support often differs between men and women [17]. Therefore, the aims of this study were to investigate which components of social support influence glycemic control and to assess if the relationship between those components and glycemic control differs between men and women with T2DM.

## Materials and methods

### Sample population

From the Medical University of South Carolina and the Ralph H. Johnson VA Medical Center, two primary care clinics in the Southeast United States, 615 adults were recruited Eligibility included age of 18 years and older, a diagnosis of diabetes documented in the medical record, and the ability to communicate in English. Ineligibility included a determination by the research coordinator that the participant had dementia, cognitive impairment, or active psychoses, such that they could not complete the survey. Based on a review of the medical records, potentially eligible patients were sent letters of invitation and approached in the waiting room during medical visits, where they were provided a detailed explanation of the study. After a detailed explanation of the study procedures, each patient who was eligible and expressed interest provided written consent to participate in this one-time research study and were administered validated questionnaires capturing social determinants of health and diabetes self-care information. Surveys included in the questionnaire were based on the conceptual framework developed by Brown et al. regarding the influence of social determinants of health on diabetes outcomes [19]. After completion of the survey, research coordinators abstracted clinical data from the participants’ medical record, including glycosylated hemoglobin A1c (HbA1c), blood pressure, weight, and height. This research was approved by the Institutional Review Board (IRB) at the Medical University of South Carolina and by the Health Services Research and Development Committee at the Ralph H. Johnson VA Medical Center. All study procedures were in compliance with ethical practices for human subjects research established by these entities.

### Variables

#### Demographic characteristics

Previously validated questionnaires from the National Health Interview Survey (NHIS) were used to collect demographic information including age, race/ethnicity, gender, marital status, annual household income, educational level, employment status, diabetes duration, and comorbidity status [20]. In addition, health status was assessed and based on self-reported/self-rated data [20].

#### Social support

Social support was assessed using the Medical Outcomes Study (MOS) Social Support survey, a 19-item scale measuring multiple dimensions of functional social support: (1) emotional/informational support—the expression of positivity, understanding, and encouragement of feelings/the provision of advice, information, guidance, and feedback; (2) tangible support—the provision of materials and assistance with behaviors; (3) affectionate support—the expression of love and affection; and (4) positive social interaction—the companionship of others to do fun things with [21]. Individual questions were rated on a 5-point Likert scale. Each measure was reliable, having an alpha >0.91.

#### Gender

Participants self-reported gender, which was used in stratified models to investigate gender differences.

### Outcomes

#### Diabetes self-care

The 11-item Summary of Diabetes Self-Care Activities (SDSCA) scale was used to assess diabetes self-care [22]. Management in four domains were assessed using the SDSCA: (1) diet—general diet and specific diet, (2) exercise, (3) blood glucose testing, and (4) foot care [22]. Scores were determined by calculating the mean number of days per week (out of 7 days) that the activity was performed. Except for specific diet, average inter-item correlations were high (mean = 0.47), and test-retest correlations were moderate (mean = 0.40) [22]. Overall, the SDSCA demonstrated adequate internal and test-retest reliability and validity making it generalizable to various populations with diabetes [22].

#### Glycemic control

HbA1c levels were abstracted from the electronic medical record for values within the 6 months prior to survey administration; this timepoint was used to increase the likelihood of identifying individuals who recently received care.

### Statistical analysis

**Fig 1:**
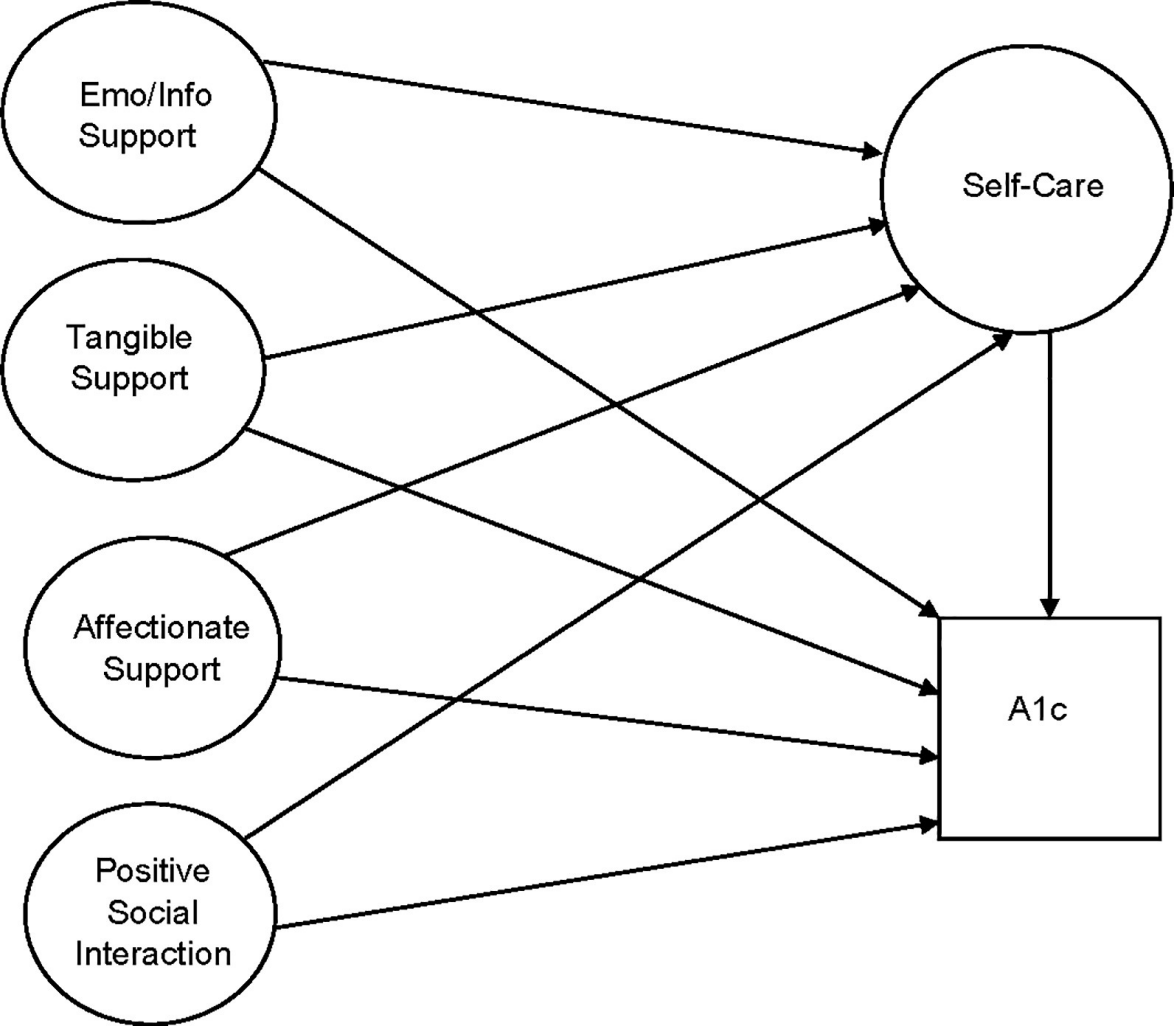
Theoretical model for the relationship between social support and glycemic control. Structural equation modeling (SEM) was used to understand pathways between components of social support and diabetes self-care and glycemic control (i.e., hemoglobin A1c).

Structural equation modeling (SEM) was used to understand pathways between social support and glycemic control based on a theoretical model, that components of social support would have an influence on diabetes self-care behaviors and glycemic control (Fig 1). SEM allows estimation of a number of closely related predictors with multiple outcomes, and combines regression, path analysis, and factor analysis to estimate both measured and latent constructs [23–26]. SEM incorporates both a measurement model, as defined in factor analysis, for the variables that load onto each latent construct, and a structural model, which estimates the relationship between variables in the model [24–26]. Models were based on a priori specifications, and analyzed following procedures outlined by Kline [24] and Acock [27].

A sample size of 615 adults was sufficient to ensure a 20:1 ratio (subject: variables) recommended to maintain 80% power in structural equation modeling [25, 28]. This sample size is also necessary to ensure stability of parameter estimates when conducting group comparisons [27]. All analyses were conducted using Stata version 14 [29].

First, a series of descriptive statistics were performed to describe the population, and investigate the normality of variables, and correlations between constructs. Data met multivariate normal and linearity assumptions [30]. Second, a confirmatory factor analysis model was estimated to identify the four social support latent factors. Third, a series of SEM models were conducted. All SEM models were conducted using a maximum likelihood estimation procedure and the ‘mlmv’ option in Stata v14, which keeps variables in the model rather than allowing listwise deletion of any missing variables. Standardized parameters were used to allow comparison between models, so parameters are interpreted as the change in standard deviation of an outcome due to one standard deviation of the predictor. Eight SEM models were run in all: (1) SEM for men and women to investigate the relationship between four social support components, self-care, and glycemic control, (2) SEM model investigating gender invariance with equality constraints on all loadings, error variances, intercepts, and covariances, (3) SEM model investigating gender invariance with no equality constraints, (4) SEM model investigating gender invariance with only loadings constrained as equal, and allowing invariance in error, intercept, and covariance, (5) SEM model investigating gender invariance with loadings and error variances both constrained, (6) SEM model investigating gender invariance with loading, intercept, and covariance constrained, (7) SEM model investigating gender invariance with loading and intercept constrained, and (8) SEM model allowing differences in means of individual responses between men and women. Models were then compared using AIC and likelihood ratio test. If more constrained models were not significantly worse than unconstrained models, the constrained model was considered preferred, and differences between genders were considered not significant on that aspect of the model.

## Results and discussion

Table 1 shows the sample demographics of adults with diabetes. Approximately 65% of the sample was Non-Hispanic Black, and 38% was women. Half of the population was married, and 52% had an annual household income <$25,000. The average age and educational attainment were 61 years and 13 years, respectively. The average number of hours worked per week were approximately 13, and the average duration of a diabetes diagnosis was 12 years.

**Table 1:** Sample demographics (n = 615).

Age (years)	61.3±0.9
Education (years)	13.4±2.8
Employment (hours per week)	12.5±19.0
Diabetes Duration (years)	12.3±9.1
Charlson Comorbidity Score	25.7±2.2
Health Literacy	26.1±10.2
Race/Ethnicity	
Non-Hispanic White	33.0
Non-Hispanic Black	64.9
Other	2.1
Gender	
Women	38.4
Men	61.6
Marital Status	
Never married	11.2
Married	49.7
Separated/Divorced	28.2
Widowed	10.9
Annual Household Income	
≤$9,999	20.2
$10,000-$14,999	11.3
$15,000-$19,999	10.1
$20,000-$24,999	10.4
$25,000-$34,999	14.7
$35,000-$49,999	13.8
$50,000-$74,999	10.1
≥$75,000	9.4
Insurance Coverage	
No Insurance	9.3
Private Insurance	20.2
Medicare	24.7
Medicaid	10.2
VA Insurance	23.9
Other	11.7
Self-Reported Health Status	
Excellent	1.3
Very Good	12.0
Good	38.2
Fair	38.7
Poor	9.8
Clinical Indicator	
Hemoglobin A1c	7.9±1.8
Self-Care Behaviors	
General diet	4.7±2.0
Special diet	4.0±1.6
Exercise	2.6±2.2
Blood glucose testing	4.6±2.5
Foot care	4.3±2.5
Site	
Academic Medical Center	51.2
Veteran Affairs Medical Center	48.8

All numbers represent mean ± standard deviation or percentage.

Table 2 shows the descriptive characteristics of the SEM factors, which include the mean values and standard deviations for social support, glycemic control, and the self-care behaviors. Mean values for the social support factors ranged from 3.7 to 4.2. Average HbA1c for the sample was 7.9%. Values for the self-care behaviors range from 2.6 (exercise) to 5.9 (medication adherence).

**Table 2:** Descriptive characteristics of structural equation model factors.

Factors/Measured Variables	Mean Values ± Standard Deviation
Social Support	
MOS-1	4.0±1.2
MOS-2	3.8±1.2
MOS-3	3.9±1.2
MOS-4	3.9±1.3
MOS-5	3.8±1.2
MOS-6	3.7±1.4
MOS-7	3.7±1.4
MOS-8	3.8±1.3
MOS-9	3.8±1.4
MOS-10	4.0±1.3
MOS-11	4.0±1.4
MOS-12	3.9±1.4
MOS-13	4.1±1.2
MOS-14	4.2±1.2
MOS-15	4.0±1.3
MOS-16	4.0±1.2
MOS-17	3.9±1.3
MOS-18	3.9±1.3
Glycemic Control	
Hemoglobin A1c	7.9±1.8
Self-Care Behaviors	
Medication Adherence	5.9±2.0
General Diet	4.7±2.0
Special Diet	4.0±1.6
Exercise	2.6±2.2
Blood Glucose Testing	4.6±2.5
Foot Care	4.3±2.5

Mean and standard deviation of each variable used to create latent variables.

Abbreviation: MOS = Medical Outcomes Study

Table 3 shows correlations for social support, glycemic control, and self-care behaviors. Glycemic control was significantly correlated with general diet (r = -0.12), exercise (r = -0.10), blood glucose testing (r = 0.09), and medication adherence (r = -0.20).

**Table 3:** Pairwise correlations for social support, glycemic control, and self-care behaviors.

	1	2	3	4	5	6	7	8	9
1. HbA1c	--	--	--	--	--	--	--	--	--
2. PCS QOL	-0.05	--	--	--	--	--	--	--	--
3. MCS QOL	-0.06	-0.18*	--	--	--	--	--	--	--
4. General diet	-0.12*	-0.06	0.14*	--	--	--	--	--	--
5. Specific diet	-0.07	-0.01	0.13*	0.36*	--	--	--	--	--
6. Exercise	-0.10*	0.04	0.18*	0.29*	0.15*	--	--	--	--
7. Blood sugar testing	0.09*	-0.02	0.00	0.21*	0.19*	0.11*	--	--	--
8. Foot care	0.03	-0.02	-0.02	0.22*	0.22*	0.12*	0.28*	--	--
9. Medication adherence	-0.20*	-0.02	0.21*	0.28*	0.26*	0.13*	0.17*	0.23*	--

*p<0.05. Abbreviations: HbA1c = Hemoglobin A1c; PCS = Physical Component Score; MCS = Mental Component Score

QOL = Quality of Life

**Fig 2:**
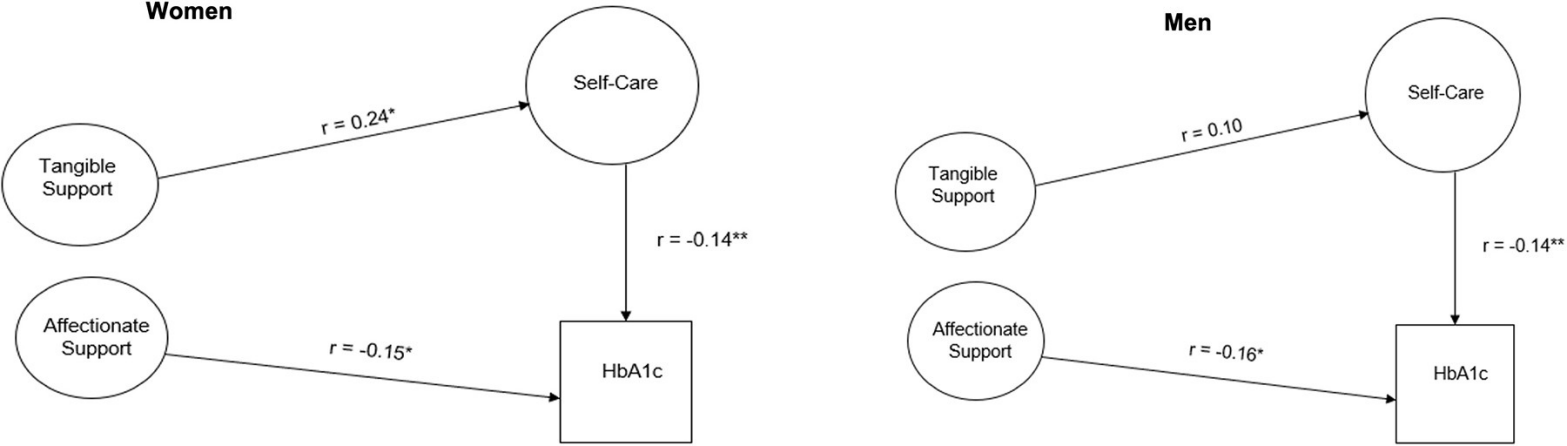
Gender invariance models between social support and glycemic control for women and men. Coefficients are standardized path coefficients. Overall model fit: Chi2 (903) = 2408.91, p b 0.001; R2 = 0.93, RMSEA = 0.052, CFI = 0.900). Statistically significant differences at *p<0.08, **p<0.05, ***p≤0.01. HbA1c = Glycosylated Hemoglobin A1c.

Tables 4 and 5 show the summaries of multiple group comparisons. In the model for men and women (Model 1 from methods), tangible support was significantly associated with self-care (r = 0.16; p = 0.046) and affectionate support was marginally associated with glycemic control (r = 0.15; p = 0.08). Using SEM to test gender invariance (Models 2–7 from methods), there was no statistically significant difference in the meaning of social support between men and women. Both men and women incorporate all four components in their latent construct of social support (Model 3 compared to Model 2 was not significantly worse), and each latent component has the same meaning (Model 4 compared to Model 3 was not significantly worse). There were different unique variances in the response of women and men to individual items measured in the social support questionnaire (Model 5 compared to Model 4 was significantly worse, LR<0.001). There were differences in means of latent variables, indicating mean emotional support for men was marginally statistically lower than for women with a small effect size (r = -0.14, p = 0.09 in Model 8). Unique invariance in responses also showed a stronger relationship between tangible support and self-care for women than for men (r = 0.24; p = 0.06 in Model 8) (Fig 2).

**Table 4:** Model comparisons.

Model	Chi-squared (df)	Comparison	Chi-squared (df) difference
1. Same form model	1099.50 (512)		
2. Equal loading model	1123.53 (536)	2 vs. 1	24.03 (24), p = 0.46
3. Equal loading and errors	1240.51 (566)	3 vs. 1	141.01 (54), p<0.001
		3 vs. 2	116.98 (30), p<0.001
4. Equal loading, errors, variances, and covariances	1167.19 (571)	4 vs. 1	67.69 (59), p = 0.20
		4 vs. 2	43.66 (35), p = 0.15
		4 vs. 3	-73.32 (5), p = 1.0
5. Equal loading and intercept	1156.31 (561)	5 vs. 1	56.81 (49), p = 0.21
		5 vs. 2	32.78 (25), p = 0.14
		5 vs. 3	84.20 (5), p<0.001
		5 vs. 4	10.88 (1), p = 0.37
6. Comparison of means	1147.55 (557)		

**Table 5:** Summary table for multiple group comparisons.

	Unconstrained		Constrained	
	Men	Women	Men	Women
**Self-Care Behaviors**				
→ Emotional Support	0.10	0.10	0.13	0.04
→ Tangible Support	0.16*	0.16*	0.10	0.24*
→ Affectionate Support	0.03	0.03	0.04	0.04
→ Positive Interaction	0.11	0.11	0.12	0.10
**Glycemic Control**				
→ Self-care	-0.14**	-0.14**	-0.14**	-0.14**
→ Emotional Support	-0.08	-0.08	-0.08	-0.08
→ Tangible Support	-0.03	-0.03	-0.03	-0.03
→ Affectionate Support	0.15*	0.15*	-0.16*	-0.15*
→ Positive Interaction	-0.09	-0.09	-0.09	-0.09

* p<0.08

** p<0.01

*** p<0.001

Of the four components of social support, tangible and affectionate support had the strongest influences on glycemic control. While men and women have the same structural representation and meaning for each of the four components of social support, there were gender differences in the relationship between these components and diabetes self-care and outcomes. Based on these results, attention to affectionate support will help improve glycemic control in both men and women, whereas, providing tangible support may help improve self-care management, particularly among women. In summary, these findings suggest the development of future interventions that include components of social support, specifically affectionate support targeted at improving glycemic control in both men and women with T2DM, and tangible support tailored to improve self-care behaviors in women with T2DM.

In our study, we found men and women with diabetes to share the same meaning of social support, and tangible and affectionate support to be the components of social support most associated with diabetes-related outcomes. Specifically, tangible support was associated with improved self-care for both men and women, but more so for women. In a study to determine the types of social support predictive of daily activities among older adults without diabetes, Hand et al found greater perceived tangible support as a type of support strongly associated with participation in daily activities [13]. Similarly, Hernandez et al found tangible support as a type of support useful in preventing or delaying the onset of cardiovascular disease among a large sample of Black adults [31]. In our study, affectionate support was marginally associated with glycemic control in men and women. While previous studies have examined the relationship between tangible support and diabetes-related outcomes, studies assessing the unique relationship between affectionate support and diabetes are limited [13]. Finally, while marginally significant, men had lower emotional support compared to women. In a study to assess the effects of gender and gender role identification on support seeking, Ashton & Fuehrer found men were less likely to seek emotional support when needing help compared to women [16]. This finding may be due to differences in socialization and personality, as femininity in both sexes has been associated with seeking and receiving emotional support, while masculinity in both sexes has been linked with receiving tangible support [32]–a finding that was more strongly associated with improved self-care behaviors among women in our study.

While the various mechanisms directly and indirectly influencing the relationship between social support and glycemic control remain indeterminate, positive and constructive social interactions can reasonably be considered as facilitators underlying the process. Evidence suggests illness-specific support often results in more favorable outcomes and predictive health behaviors compared to general or global support [9]. In diabetes, social network members such as family members, friends, providers, and peers often provide various types of positive assistance including emotional, informational, and tangible that directly or indirectly influence self-management practices and result in desired outcomes [9]. High levels of emotional support, for example, have been associated with improved lifestyle behaviors [7]. Similarly, self-efficacy, defined as a confidence in abilities to perform specific behaviors to achieve specified goals, can mediate the relationship between social support and self-management, resulting in improved glycemic control [9, 33–36]. Self-efficacy has been shown to promote healthy behavior change, improve decision making, and foster well-being [35]. Compared to men with diabetes, women with diabetes have been shown to have higher levels of self-efficacy, leading to better diabetes-related outcomes [35] Finally, in combination with self-efficacy, increased access to care serves as a mechanism by which social support protects against the physiologic response of stress [6]. This results in a positive relationship between social support and glycemic control and results in a decreased risk of mortality for adults with diabetes [6].

This study has limitations that are worth mentioning, First, the data are cross-sectional, which limits the ability to infer causality or direction of effect. While the methodology used to analyze data in this study is useful for elucidating pathways, additional research using longitudinal data is warranted to identify direction of effect. Second, this study was conducted in adults residing in the southeastern United States; therefore, the generalizability of these findings to adults living in other geographic locations may be limited. Future research elsewhere is needed to validate these study findings. Third, one questionnaire was identified and used to assess diabetes self-care activities in this sample; the same was done to assess social support. Alternative tools for assessing these variables could have yielded different results. Fourth, the research assistants determined whether individuals were able to complete the survey instruments, which could have introduced selection bias. Future research should include projects where study participants are randomized into groups to allow for comparison between individuals with similar characteristics. Finally, additional factors can influence the latent and measured variables assessed in the study and may further explain relationships and underlying mechanisms. Additional studies should account for these confounding factors.

## Conclusions

The results of our study are important and provide new information about gender differences in the relationship between social support and diabetes-related outcomes. Components of social support, specifically affectionate and tangible support, were found to influence glycemic control and self-care by gender. Specifically, in this sample of adults with T2DM, the meaning of social support did not differ between men and women; however, affectionate support was associated with improved glycemic control in both men and women, and tangible support was associated with improved self-care behaviors in women. These findings point to the need for tailored interventions utilizing affectionate and tangible support to improve clinical and behavioral outcomes in adults with T2DM.

## Supporting information

S1 File (DOCX)

S2 File (DOCX)
