# Supplementary material for: Gender invariance in the relationship between social support and glycemic control
Source: PLoS One. 2023 May 8;18(5):e0285373. doi: 10.1371/journal.pone.0285373 (PMC10166517; doi:10.1371/journal.pone.0285373)
Supplement: S1 File — (DOCX) [file pone.0285373.s001.docx]

**Author Contributions**

**Leonard E. Egede:** funding acquisition, conceptualization, data curation, formal analysis, methodology, writing – review & editing

**Rebekah J. Walker:** data curation, formal analysis, methodology, writing – original draft preparation, writing – review & editing.

**Joni S. Williams:** writing – original draft preparation**,** writing – review & editing.

**Conflict of Interest**

The authors report no potential conflicts of interest relevant to this article.

**Funding**

This study was supported by Grant K24DK093699-01 from the National Institute of Diabetes and Digestive and Kidney Diseases (PI: Leonard E. Egede, MD, MS)

https://www.niddk.nih.gov/

“The funders had no role in study design, data collection and analysis, decision to publish, or preparation of the manuscript.”
