# Supplementary material for: Gender invariance in the relationship between social support and glycemic control
Source: PLoS One. 2023 May 8;18(5):e0285373. doi: 10.1371/journal.pone.0285373 (PMC10166517; doi:10.1371/journal.pone.0285373)
Supplement: S2 File — (DOCX) [file pone.0285373.s002.docx]

**Study Highlights**

- Social support has the same meaning among men and women with diabetes.
- In men and women, tangible support and affectionate support had the most influence on diabetes-related outcomes.
- Tangible support is significantly associated with self-care behaviors, while affectionate support is marginally associated with glycemic control.
- Gender invariances exist in the relationship between social support and glycemic control in adults with diabetes, such that tangible support for self-care was strongest among women with diabetes.
- The use of tangible and affectionate support in future interventions may prove useful in improving diabetes-related outcomes.
